# AMH producing purely cystic virilizing adult granulosa cell tumor in 17 years old girl: a case report and review of literatures

**DOI:** 10.1186/s13048-023-01134-0

**Published:** 2023-03-15

**Authors:** Michio Kitajima, Itsuki Kajimura, Yuriko Kitajima, Naoko Murakami, Asako Matsumura, Kanako Matsumoto, Ayumi Harada, Yuri Hasegawa, Kiyonori Miura

**Affiliations:** 1 Department of Obstetrics and Gynecology, Nagasaki University Graduate School of Biomedical Sciences, 1-7-1 Sakamoto, Nagasaki, 852-8501 Japan; 2 Department of Obstetrics and Gynecology, Nagasaki University Hospital, 1-7-1 Sakamoto, Nagasaki, 852-8501 Japan

**Keywords:** Cystic granulosa cell tumor, Hyperandrogenism, AMH

## Abstract

**Background:**

Androgen-producing granulosa cell tumor in adolescent girl is rare condition and clinical characteristics are not fully elucidated.

**Case presentation:**

Seventeen years old girl complained of secondary amenorrhea was referred to our out-patient consultation. Markedly elevated serum testosterone, LH, and AMH levels were noted. Mild hirsutism and clitoromegaly were presented. Transabdominal ultrasonography and MRI revealed cystic mass occupied pelvic cavity probably originated from left ovary. Right ovary showed polycystic appearance. Laparoscopic left ovarian cystectomy was performed. After the surgery, her menstruation resumed along with normalized hormonal parameters, and clinical hyperandrogenism were improved. Since the scarcity of cellular lining of inner cyst wall, definitive pathological diagnosis was difficult. After the consultation with gynecological pathologist, the tumor was diagnosed as sex cord stromal tumor, highly suspicious for adult granulosa cell tumor. Residual left salpingo-oophorectomy was performed by additional laparoscopic surgery. Her serum testosterone and AMH levels were remained low with regular menstrual cycles and no evidence of recurrence.

**Conclusions:**

Androgen-producing cystic granulosa cell tumor is rare gynecological disorders, which need both gynecologic oncological and endocrinological approach. Its clinical manifestations may bring some clues to the pathogenesis of ovulatory dysfunctions, such as polycystic ovary syndrome.

## Background

Sex cord stromal tumor of the ovary is a rare condition comparing to other histological type of ovarian tumor. Granulosa cell tumor (GCT) is the most common malignant sex cord-stromal tumor and constitutes 3–5% of all ovarian malignancies [1]. GCTs are divided into two histopathological subtypes, classified as adult-type and juvenile-type. The adult-subtype representing 95% of all GCTs, develop in perimenopausal or postmenopausal women, at a peak age frequency between 50 and 55 years. The juvenile-type GCT is represented in 5% of cases, mostly recognized in the prepubertal age, at a peak age of 13 [2].

The tumor is generally described as solid masses with a variable amount of cystic component, often accompanied by hyperestrogenism [3]. Although it is rare, androgen producing GCT that provoke virilization had been reported [4–16]. The most common clinical manifestation of hyperandrogenism in these cases are amenorrhea, hirsutism, deepening of the voice, increased abdominal size, and male type distributions of pubic hair. The clinical manifestations in reported cases are vary in age, degree of virilization, tumor morphology, and endocrinological profiles. In particular, cystic GCTs may be related to androgen producing subtype and the histopathological diagnosis of cystic GCTs are sometimes challenging [17].

Anti-Mullerian hormone (AMH) is a dimeric glycoprotein produced solely from granulosa cells of the ovary in women at reproductive age [18]. In addition to its wide application in infertility care, such as determination of ovarian reserve, serum AMH levels may be useful in diagnosis of GCTs [19]. However, the information regarding AMH levels in androgen producing GCT is scarce. Recently, a case of 35 years old infertile women previously diagnosed as polycystic ovary syndrome (PCOS) showed multilocular cystic tumor with some solid portion that was diagnosed as adult type GCT with elevated AMH and hyperandrogenism that was diagnosed as having had been reported [4]. However, the role of AMH in diagnosis and clinical manifestations of androgen producing GCTs in younger age are not fully understood.

Here, we report a 17 years old girl with purely cystic GCT presenting secondary amenorrhea and virilization with elevated testosterone, LH and AMH.

## Case presentation

**Fig. 1:**
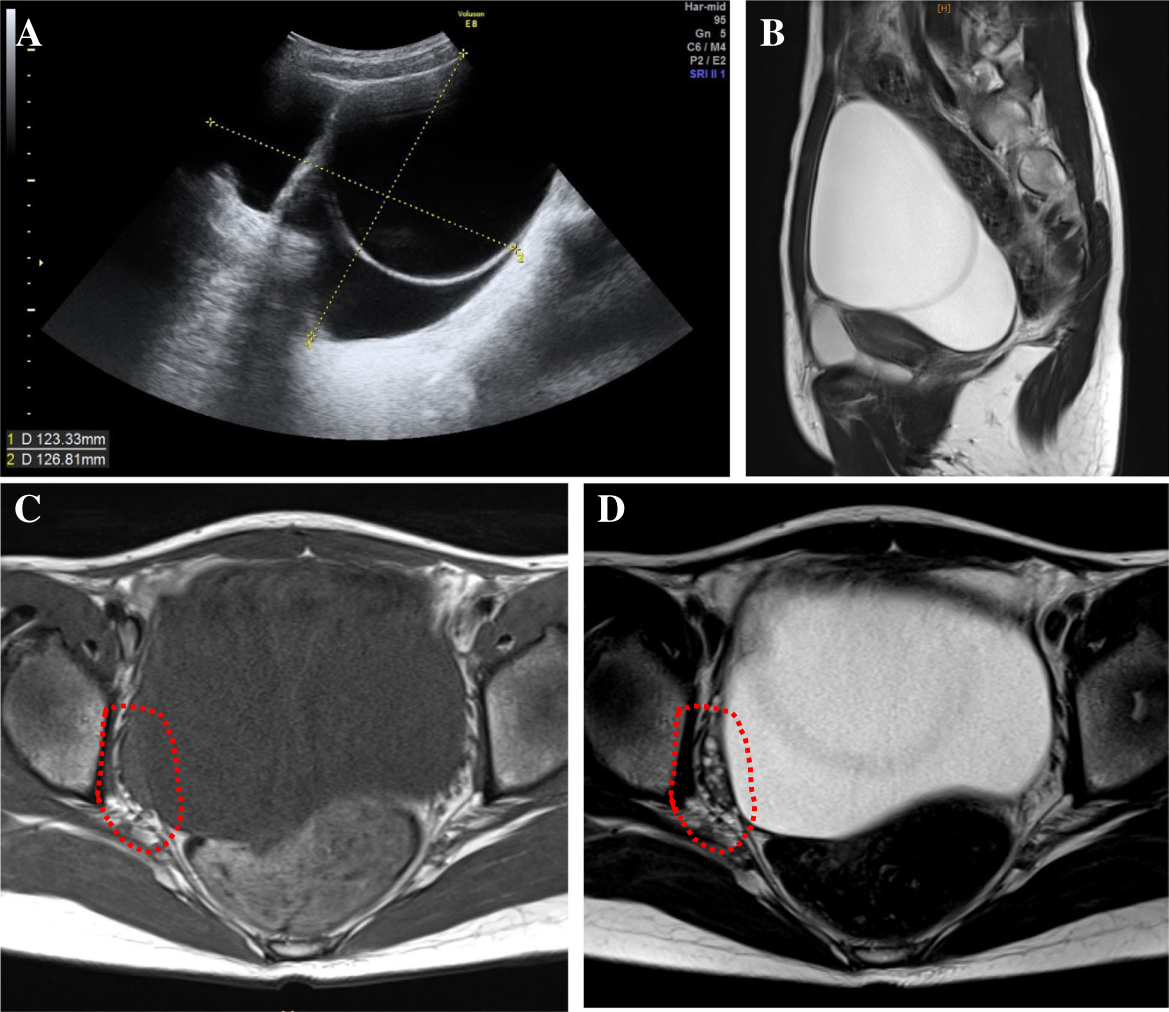
**A** Transabdominal ultrasonography showed multilocular cystic mass over 12 cm in diameter in pelvis. The wall of the cyst was thin and contents of each cyst were aechoic. **B** MRI T2 weighted sagittal image; multilocular cystic mass without any solid part occupied in pelvis. **C** and **D** MRI T1 (**C**) and T2 (**D**) weighted transverse image may indicated serous fluid contents of the cysts. Dotted circles indicate right ovary, which is polycystic appearance

**Fig. 2:**
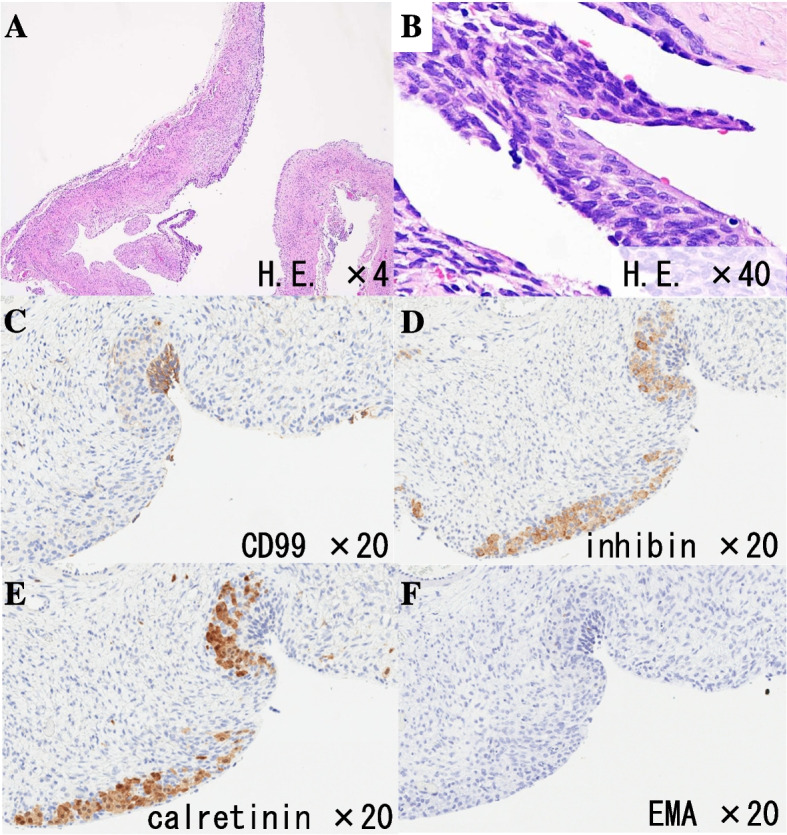
**A** and **B** Hematoxylin and Eosin staining. **A** cyst wall (low power view): Fibrous connective tissue of the cyst wall with defect of inner cellular lining in many parts. Focal portion of stratified cellular lining with enlarged chromatic nucleous was noted. **B** cyst wall (high power view): Cells resembling granulosa cell with coffee bean like nuclear grooves were noted. **C**-**F** Immunohistochemistry. **C** CD99. **D** inhibin. **E** calretinin. **F** EMA (epitelial membrane antigen)

Seventeen years old girl complained of secondary amenorrhea were referred to our out-patient consultation. Her menarche was at 14 years old and 30 days regular menstrual cycle were maintained until 16 years old. On general and gynecological examination, mild hirsutism at face, neck and pubis and mild clitoromegaly were noted. Blood examination revealed markedly elevated serum LH, testosterone, and AMH levels (Table 1). Transabdominal ultrasonography revealed pelvic cystic mass probably originated from left ovary (Fig. 1A). Pelvic MRI confirmed left ovarian cystic tumor resembling serous adenoma without any solid mural nodules nor thickening of the septum (Fig. 1B). There is no other tumor lesion in peritoneal cavity. Accumulation of small follicles in right ovary, which resemble polycystic ovary were noted (Fig. 1C and D). Serum markers for epithelial ovarian tumor, such as CA125, CA19-9 and CEA, were all under the threshold levels. Under the suspects of hormone producing ovarian tumor, we performed laparoscopic left ovarian cystectomy. In the pelvic cavity, serous yellowish colored ascites was noticed. The cytology of the ascites was benign. Using S.A.N.D. balloon catheter (Hakko, Tokyo), cyst was punctured, and yellow-colored serous contents of cystic tumor were aspirated. Left ovary was pulled and cystectomy was performed via extracorporeal manipulation. Excised left ovary was approximated with absorbable suture. Post operative course was uneventful. After the surgery, her menstruation resumed along with normalized hormonal parameters (Table 1). Clinical hyperandrogenism were improved. Histopathologial analysis of cyst wall revealed thin fibrous connective tissue with defects of inner cellular lining in many parts (Fig. 2A). Focal portion of stratified cellular lining with enlarged chromatic nucleus was noted. These cells are resembling granulosa cell with coffee bean like nuclear groove (Fig. 2B). Immunohistochemical analysis of inner cellular lining with CD99 (focally positive), inhibin (positive), calretinin (positive) and epithelial membrane antigen (EMA) (negative) may agree with granulosa cell tumor (Fig. 2C-F). Due to the scarceness of inner cellular lining among the cyst wall, definitive pathological diagnosis was difficult. After the consultation with gynecological pathologist, the tumor was diagnosed as sex cord stromal tumor, highly suspicious for adult granulosa cell tumor. After the multi-disciplinary consultation including patient and her family, additional laparoscopic surgery to excise residual left ovary and tube (salpingo-oophorectomy) was performed at 12 months after the primary surgery. laparoscopic exploration of abdominal cavity including peritoneal and omental biopsy and cytology of peritoneal fluids were performed. There was no residual tumor lesion grossly nor microscopically, and she was diagnosed as stage IA. Her serum testosterone and AMH levels were remained low with regular menstrual cycles and no evidence of recurrence in five years follow up.

**Table 1:** Hormonal profiles before and after surgery

	Before surgery	3 weeks post surgery	3 month post surgery menstruation resume^a^	9 month post surgery regular menstruation^b^
LH (mIU/mL)	26.89	9.18	10.43	3.86
FSH (mIU/mL)	1.75	5.91	4.47	3.38
PRL (ng/mL)	15.54	34.29	17.6	31.11
P4 (ng/mL)	0.51	0.30	0.06	1.35
E2 (pg/mL)	23.7	80.2	184.3	40.9
T (ng/dL)	238.6	22.7	33.6	19.5
AMH (ng/mL)	76.5	2.17	3.58	5.22

*PRL* Prolactin, *P4* Progesterone, *E2* Estradiol, *T* Testosterone, *AMH* Anti-Müllerian hormone

^a^assay was performed in proliferative phase

^b^assay was performed in luteal phase

## Discussion and conclusions

Granulosa cell tumor (GCT) is the most common type of sex cord stromal tumor of the ovary. Due to its ability of hormone production, most of the cases (97–98%) may show hyperestrogenism, such as amenorrhea, dysfunctional menstrual bleeding, growth of uterine leiomyomas, hyperplasia of the endometrium, or endometrial cancer. In rare circumstances, androgen producing GCTs may be present (2–3%). The symptoms and signs of the virilizing GCTs are primary or secondary amenorrhea, hirsutism, clitoromegaly, deepening of the voice, muscular development and acne due to elevated testosterone levels [12]. In our case, apparent hyperandrogenism was present and they were disappeared after removal of cystic mass of left ovary.

Among the case reports of androgen producing GCTs, the degree of virilization and clinicopathological findings showed wide range of variations [4–16]. In the report of large series of virilizing GCTs, although the solid tumor is more common in GCTs, seven tumors among 17 cases were cystic, of which five with unilocular and two cases presented multilocular cystic tumor. In these seven cases, six tumors were diagnosed as adult type GCT [10]. On the other hand, androgenic juvenile type GCT with unilocular cystic presentation in 13 years old girl was also reported [11].

As GCTs are generally described as unilateral solid masses with a variable amount of cystic component, purely cystic granulosa cell tumor is rare condition. Mulvany and Riley reported six cases of cystic granulosa cell tumor, they are all larger than 10 cm and adult type, which is concord with present case [20]. In some case with virilizing GCTs, cystic form without apparent solid potion had been reported [10]. Although it is rare, estrogen producing cystic GCT in 18 years old girl that histopathologically diagnosed as jeuvinile type CGT was reported [21]. Recently, Boyraz B, et al., reported large case series of cystic GCTs [17]. They found a tendency for cystic adult GCTs to occur in younger patients than its typical counterpart, and 25% of them exhibited androgenic manifestations. In addition, cystic juvenile GCTs are more often androgenic than the prior literature has indicated (52% in their report). They also pointed out that adult or juvenile GCTs that are strikingly cystic, and extensive denudation of cyst linings in cystic GCTs often cause a diagnostic challenge. They also discussed multicystic formation of adjacent part of the lesion may be hormonally driven rather than neoplastic in nature. The possible association between cyst formation, stromal luteinization, and subsequent androgenic manifestations may be presented.

FOXL2, Forkhead box L2, belongs to the large family of forkhead/winged-helix transcription factors, and its mutation is reported to be involved in the development of adult GCTs [22]. Therefore, detection of the expression of FOXL2 in GCTs can be helpful in the diagnosis of GCTs [23]. In present case, we could not explore FOXL2 mutations due to the limited amount of cellular portion. The usefulness of FOXL2 examination in the diagnosis of androgenic cystic GCTs may need further investigations.

Since there is non-specific image study and difficulty in histological determination of purely cystic androgenic GCTs, other diagnostic measures may be desirable. AMH is a dimeric glycoprotein that is secreted solely by ovarian granulosa cells in women [18]. Studies suggested AMH may be a marker of tumor recurrence, progression, and treatment efficacy in adult type GCT [24, 25]. Reliable correlation between serum AMH levels and tumor mass that determined by pathological specimens or imaging measures had been reported [26]*.* Recent meta-analysis concluded that serum AMH can diagnose GCT with high accuracy (sensitivity:0.89 and specificity:0.93) [19]. In our case, serum AMH is markedly elevated and decreased to normal levels and maintained low after surgery without any signs of recurrence. As the adult-subtype representing 95% of all GCTs, occurs in perimenopausal or postmenopausal women, at a peak age frequency between 50 and 55 years, which age may show undetectable serum AMH without tumor, AMH may possess ideal diagnostic role. On the other hand, adolescent like our case may show high AMH levels, especially case with ovulatory dysfunction, which may limit the efficacy of serum AMH levels as a diagnosis measure. However, serum AMH levels found in women with GCTs may show well above those of women with ovulatory dysfunction even in women with cystic GCTs like our case. The role of AMH as a marker of cystic androgenic GCTs may need further investigation.

Our presented case showed hormonal profiles corresponding to hyperandrogenism and amenorrhea. Elevated testosterone and AMH may be produced from the tumor. These hormones may influence the status of contralateral ovary. In our case, contralateral ovary showed polycystic appearance. It is well known that PCOS is manifested by elevated testosterone, LH, and AMH. Elevated testosterone may stimulate LH secretions, which may stimulate theca cell activity in contralateral ovary and results in arrest of follicular growth and additional secretion of testosterone and AMH from contralateral ovary. These vicious cycles may affect morphology of contralateral ovary and acute hyperandrogenism in present case. The surgical removal of the tumor normalized hormonal profiles. On the other hand, androgen producing GCTs complicated with PCOS had been reported [4–6]. Clinical signs of PCOS may be overlapped with those of androgen producing GCTs, though apparent virilization found in GCTs may be less frequent in PCOS. In reported case, the symptom related to PCOS have not improved after the cystectomy [5]. Persistence of androgenic symptom after the surgery also reported by the others [15]. Considering the experience with our case and other reported cases, androgenic GCTs may stimulate latent PCOS status in affected women. Postsurgical follow up of ovulatory status together with morphological appearance of contralateral ovary may be recommended.

The mechanism of androgen dominant secretion by cystic GCTs are not fully understood. Altered expression of steroid synthesis enzymes may be one of the possible mechanisms. P450 aromatase is a key enzyme to convert androstenedione to estrone and estradiol. Immunohistochemical expression of P450 aromatase were confirmed in estrogen producing GCTs [27]. The expression of P450 aromatase corresponds to specific cell morphology of GCTs, including recurrent tumors [28]. A deficiency of aromatase may suppress the conversion of androgen to estrogen, thus triggering excessive androgen accumulation. On the other hand, elevated AMH produced by GCTs may be another mechanism for elevated androgen in this case. Excess AMH may trigger an imbalance in the hypothalamic–pituitary–ovarian axis, subsequently inducing elevated LH levels and hyperandrogenism via binding to the AMH receptors of GnRH neurons [29]*.* However, the elevation of testosterone and LH can be affected by multiple pathways; therefore, the definite mechanism may need more studies.

In conclusion, cystic virilizing GCTs are rare causes of hyperandrogenism in adolescents. A definitive diagnosis by histopathology may be difficult due to denudation of cellular lining of cyst wall. Markedly elevated serum AMH levels may be useful for diagnosis. Elevated testosterone and AMH may affect contralateral ovarian status. Latent ovulatory dysfunctions may be modified by hormones secreted by the tumor. The cyst may be treated surgically; most of the cases are discovered at an early stage; without a need for postoperative adjuvant therapy; however, long-term follow up should be considered.

## Data Availability

The datasets used and/or analysed during the current study are available from the corresponding author on reasonable request.
